# Abandoned Acid? Understanding Adherence to Bisphosphonate Medications for the Prevention of Osteoporosis among Older Women: A Qualitative Longitudinal Study

**DOI:** 10.1371/journal.pone.0083552

**Published:** 2014-01-02

**Authors:** Charlotte Salter, Lisa McDaid, Debi Bhattacharya, Richard Holland, Tarnya Marshall, Amanda Howe

**Affiliations:** 1 Norwich Medical School, Faculty of Medicine & Health Science, University of East Anglia, Norwich, Norfolk, United Kingdom; 2 School of Chemistry and Pharmacy, University of East Anglia, Norwich, Norfolk. United Kingdom; 3 Rheumatology Department, Norfolk & Norwich University Foundation Trust Hospital. Norwich, Norfolk. United Kingdom; The University of New South Wales, Australia

## Abstract

**Background:**

There is significant morbidity and mortality caused by the complications of osteoporosis, for which ageing is the greatest epidemiological risk factor. Preventive medications to delay osteoporosis are available, but little is known about motivators to adhere to these in the context of a symptomless condition with evidence based on screening results.

**Aim:**

To describe key perceptions that influence older women's adherence and persistence with prescribed medication when identified to be at a higher than average risk of fracture.

**Design of Study:**

A longitudinal qualitative study embedded within a multi-centre trial exploring the effectiveness of screening for prevention of fractures.

**Setting:**

Primary care, Norfolk. United Kingdom

**Methods:**

Thirty older women aged 70–85 years of age who were offered preventive medication for osteoporosis and agreed to undertake two interviews at 6 and 24 months post-first prescription.

**Results:**

There were no overall predictors of adherence which varied markedly over time. Participants' perceptions and motivations to persist with medication were influenced by six core themes: understanding adherence and non-adherence, motivations and self-care, appraising and prioritising risk, anticipating and managing side effects, problems of understanding, and decision making around medication. Those engaged with supportive professionals could better tolerate and overcome barriers such as side-effects.

**Conclusions:**

Many issues are raised following screening in a cohort of women who have not previously sought advice about their bone health. Adherence to preventive medication for osteoporosis is complex and multifaceted. Individual participant understanding, choice, risk and perceived need all interact to produce unpredictable patterns of usage and acceptability. There are clear implications for practice and health professionals should not assume adherence in any older women prescribed medication for the prevention of osteoporosis. The beliefs and motivations of participants and their healthcare providers regarding the need to establish acceptable medication regimes is key to promoting and sustaining adherence.

## Introduction

There is significant morbidity and mortality caused by the complications of osteoporosis, for which ageing is the greatest epidemiological risk factor. While other risks such as immobility, persistent low body weight, early menopause and corticosteroid use may lead to early onset of osteoporosis, around 40% of women aged 70 will have osteoporosis, and as many as 90% will have a significantly increased risk of fracturing a bone in a fall or accident [1], [2]. This has led to a major research focus on the prevention of osteoporosis which has established that osteoporotic fractures can be significantly reduced by a combination of pharmacological (bisphosphonates with calcium and vitamin D supplements) and behavioural interventions (dietary intake, smoking cessation, and weight bearing exercise).

More recent initiatives include the development of treatment algorithms and encouragement for primary care practitioners to identify patients who may be at ‘risk’ of fracturing and may benefit from preventive options [3],[4]. However, as the National Institute for Clinical Excellence (NICE) acknowledge, *“identifying who will benefit from preventative treatment is imprecise”* [5]. No population screening programme currently exists for osteoporosis risk, and individuals are identified clinically on a case by case basis. Predictive risk of fracture compared to the norm for age and sex can now be calculated using clinical risk factors in conjunction with bone mineral density (BMD) measurements using Dual energy X-ray Absorbtiometry (DXA) scans [6], [7], creating new opportunities to identify individuals yet to sustain a fracture.

For screening to be effective, participants identified as high risk must be receptive to the intervention. Achieving long-term adherence to prescribed medications is more complex than just providing sufficient information or an acceptable medication regimen. The literature suggests adherence is highly variable in osteoporosis prevention with age and co-morbidity explaining relatively little of the variability [8], [9]. Attempts to reduce complexity in dosing regimens do not necessarily improve adherence [10], [11]. Patients are recommended to take their bisphosphonates first thing in the morning before eating or drinking and with a glass of water. There is a requirement for them to remain upright for 30 minutes to avoid irritation to the oesophagus. Calcium supplements are frequently provided in the form of chewy tablets. Patients may understand the potential for osteoporosis to have a negative effect on their lives, and express strong motivation to protect their health, but this does not always align with taking medications [12], [13]. This therefore makes the motivations and decision making of older women around uptake of preventive medication of primary importance to the public health impact of any potential screening programme as well as to the individual patient.

It is also known that patterns of adherence to osteoporosis medications vary over time [14], [15]. However, a survey of patients and physicians showed that poor adherence reflected patient scepticism about the risks and values of treatment, rather than a lack of factual knowledge. A qualitative synthesis of studies on lay experience of medicine taking found widespread caution about taking medication with many participants ‘testing’ prescribed medicines for efficacy and adverse side effects [14]. Our pilot study found as many as 50% of women at ‘high risk’ of fracture in the 70–85 age group were not receiving treatment four months later [16]. We therefore undertook this study to explore the factors that influence older women's adherence to prescribed prophylactic medication when assessed to have higher than average risk of fracture following screening. This paper describes the perceptions and motivations to which participants attributed their willingness and ability to adhere to osteoporosis prevention regimes, and considers implications for practice.

## Methods

### Participants and procedure

The Adherence To Osteoporosis Medication (ATOM Study) was established as a longitudinal qualitative study embedded within the Medical Research Council funded UK multi-centre randomised control trial on Screening for Osteoporosis in Older Women for the Prevention of Fractures (SCOOP). SCOOP [17] aims to explore the effectiveness of screening women aged 70–85 for the prevention of fractures using a risk-prediction algorithm. The qualitative study took place in Norfolk, United Kingdom.

### Ethics Statement

We secured approval from North West National Health Service Research Ethics Committee (Ref: 07/H1010/70). Written informed consent was obtained from participants. Two participants with mild cognitive impairment were supported in the consenting and interview process by their husbands.

The research comprised a longitudinal design with two in-depth interviews conducted 18 months apart, the first at around 6 months post-randomisation. The sample was drawn from those found to be at ‘higher than average’ risk of a subsequent fracture and whose prescribing data showed they had started medications for the prevention of osteoporosis. Participants were purposively sampled from demographic and adherence data already collected by the SCOOP trial (see Table 1). As the focus of our study was to explore why older women were adherent, we constructed our sample to include more women self-reporting they were adherent when contacted by phone than reporting they were non-adherent to their osteoporosis medication.

**Table 1:** Sample characteristics using pre-collected trial data.

Sample Characteristic	Category	Number (%)
Self-reported adherence status given on phone at recruitment	AdherentNon-adherent	19 (63)11 (37)
Age	70–7475–7980+	9 (30)10 (33)11 (37)
GP practice	UrbanRural	14 (47)16 (53)
Social class	IIIIIINIIIMIVV	3 (10)6 (20)6 (20)10 (33)5 (17)0

For the purpose of this study ‘adherent’ included both women stating they were taking bisphosphonate medication as instructed, and those stating they were intentionally or unintentionally missing doses but no more than 1 in 5 (i.e. 80% adherence or more). Non-adherent' included all those who had discontinued bisphosphonate medication, or were taking them <80% of the time, but who might still be taking prescribed supplements (calcium and vitamin D).

### The interviews

Interviews took place at participants' homes and lasted an average of 74 minutes. They were based on a topic guide developed to explore women's understanding of osteoporosis, responses to screening results, current usage of preventive medicine, motivators and detractors from taking medication and follow up with healthcare professionals. Interview recordings were transcribed verbatim and anonymised. Familiarisation, data management, coding and categorisation were carried out by the interdisciplinary research team including CS, LM and AH. Iteration between both data sets and the research literature helped inform the analysis at the explanatory level. The principles of Framework Analysis [18] were used to order, chart and search the data both manually and supported by relevant software (NVivo 9 Software, MSWord and Framework). Illustrative quotations are selected to elucidate the study findings. Extracts are labelled using participant number, age at interview and summary adherence status to both bisphosphonates and calcium supplements.

## Results

Ninety women in the ‘higher than average’ risk group recruited to the Norwich arm of the SCOOP Trial indicated they would be willing to take part in the qualitative sub-study. From these we recruited a sample of 30 (33%) women, age range 73–85 years (Table 1). Five participants were unable to participate in the follow-up interview due to death or withdrawal from the study.

### Understanding adherence and non-adherence

All 30 participants were prescribed bisphosphonates and all except one commenced their first course. Of the 10 participants shown in Table 2 who reported being non-adherent at Phase 1 Interviews, nine made this decision without discussion with their general pracitioner. All bar one said they had done this within a month of collecting their first prescription. The combination of bisphosphonate and calcium: vitamin D supplements was reported to be taken by 12 participants.

**Table 2:** Summary of Patterns of Adherence.

	Bisphosphonates	Calcium Supplements	Bisphosphonates & Calcium Supplements
	N (%)	N (%)	N (%)
**Phase 1**			
Adherent	20 (67)	15 (50)	12 (40)
Non-adherent	10 (33)	10 (33)	13 (43)
Not prescribed	-	5 (17)	5 (17)
Total	30 (100)	30 (100)	30 (100)
**Phase 2**			
Adherent	14 (56)	12 (48)	9 (36)
Non-adherent	11 (44)	9 (36)	12 (48)
Not prescribed	-	4 (16)	4 (16)
Total	25 (100)	25 (100)	25 (100)

Of the 25 participants who took part in Phase 2 Interviews, thirteen had remained adherent to bisphosphonate medication and one previously non-adherent participant reported she had started taking her medication as prescribed. Eleven were non-adherent including three women that had given up their bisphosphonate medicine between interviews. Thus, a significant proportion of our sample were taking *no medication* for the prevention of fracture and osteoporosis at 18 months (44%). Even within the ‘adherent’ group, many women admitted deficits in their adherence; sometimes this was deliberate, to avoid inconvenience, sometimes it was because they forgot one day, but took it the next.

We found no obvious pattern or factors linking with adherence. Responses to screening, acceptance of risk status, existing medical history, previous experience of falls, fractures and family history did not appear to predict womens' adherence status.

Some participants complained about the complexity of the regimen, many had experienced side effects, some said their general practitioner had stopped the medication, and some had misunderstood the reasons for taking them long-term. However, many adherent women reported similar issues. Few cited ‘forgetting’ as a key cause of non-adherence. Almost all respondents declared a willingness to ‘in principle’ do what their general practitioner advised, but some non-adherent women cited medical permission or support for their choice to stop:

He was quite happy, he said alright just stop. He said we've had no broken bones in your family, he said you'll probably be quite alright. (Participant 12, age 84 – became Non-Adherent to Bisphosphonates by Phase 2. Refused Calcium)

Personal scepticism about the value of the treatments did not seem to link clearly with non-adherence. For example, the following participant was adherent to her medications, but demonstrated very little belief that she needed them at her age:

I thought well yes I am 80, so I probably have anyway (thinning bones) and I also thought it is a bit *late* to start treating me now I honestly did. That was my sort of attitude but the letter said ‘go and see your doctor’, so I went and saw my doctor and he gave me those. (Participant 24, age 80 - Adherent to both Bisphosphonates & Calcium Phase 1, subsequently withdrew)

By contrast Participant 22 was non-adherent. She felt anyone could break a bone in the next 10 years and would have expected to fracture by now if she was really at risk. She described how her own mother had fallen and broken her hip yet appeared to remain personally unconcerned:

When they said well look ‘a higher risk of breaking a bone over the next 10 years’ and I thought well *I'm over 80 so it's not surprising* (laughs). (Participant 22, age 84 – Continually Non-Adherent to both Bisphosphonates & Calcium)

### Motivations, self-care and adherence

All the respondents regardless of adherence status seemed to have accepted the need for better self-care and an altered lifestyle in order to prevent fractures. Many believed they had been doing this all their lives through a good diet, plenty of physical activity and exercise:

Because I've always taken calcium you know. I've always had a lot of cheese, a lot of yoghurt and I drink a certain amount of milk I have calcium and I have a lot of vegetables. (Participant 10, age 73 - Continually adherent bisphosphonates. Calcium not prescribed)

I take a cod liver oil pill every day, winter and summer. I'm sure that's a help. (Participant 09, age 85 - Continually non-adherent bisphosphonates & calcium)

In addition, many participants had adjusted their daily routines to enhance their capacity to take their regimens as prescribed. Weekly doses were linked with memorable events, and chores such as ironing utilised to fulfil the half hour required in remaining upright:

So I try and get up early, take it with this load of water and find something to do standing up, whether it's ironing for an hour which I did this week (laughs) or going round the garden seeing what's in flower. You have just got to find something to do which takes your mind off it. (Participant 06, age 80 – Continually Adherent to Bisphosphonates. Non-adherent to Calcium)

Market day is a Wednesday and I always used to go down to buy plants every Wednesday. I always used to think I can't go down and get any plants, so I always remember Wednesday. That was my day. (Participant 30, age 75 – Continually Non-Adherent to Bisphosphonates. Took Calcium supplements at Phase 2)

Carers played a role in aiding adherence for two participants with cognitive impairments by bringing the medication to them and altering the routine to ensure no food or cup of tea at the same time:

He'd put it on his computer to remind me (and) he puts them in front of me and lets me get on with it. (Participant 17, age 75 – Continually Adherent Bisphosphonates. Calcium not prescribed).

Autonomy was also a powerful motivator, characterised by the need to be independent and responsible in order to be able to care for self and others:

Well it is just the independence. I don't want to be a nuisance to the family at all if I can help it, and if I haven't done something that might have helped I'd feel a bit guilty. If you can do anything to prevent that happening it does help a little bit. (Participant 03, age 83 – Continually Adherent Bisphosphonates. Calcium not prescribed)

I like to protect myself as much as possible for my husband, well for me (too) for me I mean obviously. (Participant 18, age 80 – Non-Adherent Bisphosphonates by Phase 2. Non-adherent to Calcium)

### Appraising and prioritising risk

Phase 1 interviews specifically asked participants about their reaction to their recent risk assessment. Risk perceptions at this phase were mostly expressed in ‘sense making’ comments regarding the context of ageing. There was added complexity for participants who had been given a risk status of ‘higher than average’ but had no visible signs or experience of symptoms:

*Not in a million years*. I thought oh they'll come back and say oh you're fine. And they wrote back and said I wasn't. Yes I thought I couldn't believe it because I've always had a balanced diet. (Participant 29, age 75 – Continually Adherent to both Bisphosphonates & Calcium)

Many of those who had initially questioned their risk status and expressed negative reactions had adjusted to their status and cited measures taken to be self-protective such as not climbing ladders and prioritising a calcium rich diet:

Oh yeah it's made me *more* careful since I had that density scan and had the letter to say that um (pause) you know on average, if I fell over I would more easily break a bone than you know than normal. So that was a *good* thing because it has made me more (careful) and as you notice, I've got no rugs. (Participant 24, age 80 - Adherent to both Bisphosphonates & Calcium Phase 1, subsequently withdrew)

We explored the data for links between positive self-caring attitudes (as exemplified by women in the first interviews giving examples of longstanding commitment to weight bearing exercise and good nutrition) and active embracing of pharmacological options for preventing fractures. We also looked for an interrelationship between women's ‘accepting’ versus ‘questioning’ of their risk assessment and adherence to prescribed medication, including their reported participation in the decision making process and recourse to other support and information. Neither state appeared to be linked with long-term adherence with equal numbers of women remaining adherent to their medication who were ‘questioning’ (n = 7) as ‘accepting’ (n = 7). Furthermore, there was no link to adherence from either an initial strong emotive reaction or passive acceptance. For example, the following initially adherent participant had moved from a state of shock to positive acceptance, and yet gave up on her medication within a year:

Well in a way when I got over the shock I thought well I know something more about my body. (Participant 07, age 74 - Became Non- Adherent to Bisphosphonates by Phase 2. Adherent to Calcium)

However, the long-term and hidden changes of bones which ‘thin’ or ‘crumble’ seemed a lower priority than other illnesses. Participants' recall of medication reviews mirrored this, with most women reporting that their osteoporosis medications was rarely reviewed or mentioned in consultations.

### Anticipating and managing side effects

Eighteen women experienced side effects from mild to very severe. These ranged from unsettled stomach problems to violent nausea, vomiting bile, and burning:

They're *horrible* they really were. Well I mean honestly I got a really sore stomach and then on Monday my stomach was really bad and honestly it just felt full of air and I'd touch it and it was sore. (Participant 24, age 80 - Adherent to both Bisphosphonates & Calcium Phase 1, subsequently withdrew)

While severe side effects were linked to non-adherence, there was no simple relationship between side effects and persisting with medication, some trying up to 3 different medications. In fact, the anticipation of side effects seemed to be enough to put off some participants, with three in particular reporting that they were put off by the possible side effects described in the medicines information leaflet. Even the name ‘alendronic acid’ was cause for concern for some:

Because it says *acid* (laughs). It isn't a natural thing. I just don't like the idea of taking an acid and not lying down. (Participant 13, age 75 – became adherent by Phase 2. Adherent to Calcium)

The need for support concerning side effects was also highlighted by a number of non-adherent women who anticipated side effects that might aggravate existing problems:

To me it seemed more important that I, you know, (avoid) this reflux than breaking my hip because I thought I can be *careful*. (Participant 23, age 81 – tried one Bisphosphonate tablet only. Non-starter Calcium)

The tablets that he gave me when I read the side effects it was like a *horror film* really. And I thought well I'm better off chancing, sort of breaking a bone, than all the horrible things it said on there about (how) you could get these stomach ulcers and all things like that. And I do suffer sort of with heartburn and things like that. You have to sort of keep upright for so long after taking the tablets. I thought well I'm better off as I am than more things wrong really. (Participant 21, age 75 – non-starter Bisphosphonates and Calcium)

### Problems of understanding

A number of problems of understanding were evident in the interviews regarding osteoporosis risk, prevention and management in older women. Although there was fear and concern about developing osteoporosis, there was also a perception that falling and fracturing were normal in old age. Others debated the magnitude of risk, especially when compared to problems such as diabetes and heart disease. Some women questioned why the Dual energy X-ray Absorbtiometry screening had not been repeated to monitor the effectiveness of the medication. Nine patients adherent at Phase 1 mentioned this specifically, and 3 of these had become non-adherent by Phase 2:

I really would like to have another scan to see how my body is now, if it's any worse or still the same or whatever. I'd like to do that but apparently they don't, they like to leave it a certain amount of years don't they? I've read that. (Participant 24, age 80 - Adherent to both Bisphosphonates & Calcium Phase 1, subsequently withdrew)

I mean if you're going through all that performance, well it was for me with the lack of being able to swallow, but if you think you are doing that to no avail, you think well what's the point. (Participant 25, age 77 - became Non-adherent to Bisphosphonates & Calcium Phase 2)

A number of women in both groups expressed significant confusion about the nature and importance of the risk portrayed by the positive screening result. There was also confusion between falls and fractures, with some participants talking about their fracture risk in terms of instability rather than fragility:

As I say, I would have thought somebody with osteoporosis would have fallen over and broken their bones. (Participant 07, age 74 - Non adherent to Bisphosphonates by Phase 2. Adherent to Calcium)

I was surprised because I felt that I'd not had a lot of falls or tottery or anything. I thought people that were at higher risk were inclined to fall. (Participant 01, age 75 - Continually Adherent to Bisphosphonates. Took Calcium intermittently)

So I thought well that (bisphosphonate medication) will prevent me falling cos the main reason was if my bones got stronger I could do more in the garden and things like that and get on the steps more often. I can't anyway cos I get dizzy (but) that was my thoughts. (Participant 30, age 75 - Continually Non adherent to Bisphosphonates. Took Calcium at Phase 2)

Confusion about the effectiveness of the medication was common and participants frequently reflected on ‘not knowing’ if they were getting any benefit from taking the medication or not. Participant 16 was fully adherent with no side effects, but felt it was ‘discouraging not knowing if it works’, as did others:

I suppose the fact of never having broken anything coupled with the fact there's no signal in your body is there? There's nothing that tells you that there is anything wrong, so you don't feel that there's anything wrong. (Participant 08, age 75 – Continually Adherence Bisphosphonates and Calcium)

### Decision making around medication

Overall we found clear if unpredictable narratives around medication choice, with key factors being the initial result, side effects and subsequent health service intervention of medication prescription. Decisions were reviewed in a number of situations and personal costs weighed up against perceived benefits of taking the medication. Participant 09 described how she made the decision in the light of her own understanding of her needs and the relative benefit of taking the medication. She made her final decision in consultation with her doctor:

There is no bone disease or any sign of it in our family but I said I would take it and see what happened. It didn't suit me and I said I'm not taking them anymore and he (doctor) agreed. He said ‘it's no good taking them if they upset you because your diabetes is more important’. (Participant 09, age 85 – became non adherent by Phase 2 to Bisphosphonates and Calcium)

Many of those non-adherent to bisphosphonate medication at Phase 2 had not asked for, or been offered, a change of medication (n = 10). However, five women still adherent to their medications and experiencing side effects had been back to their doctor at least once for an alternative prescription by Phase 2 (Table 3).

**Table 3:** Summary of Bisphosphonate medication changes and adherence for participants that experienced side effects at Phase 2 (n = 18).

	Adherent	Non-adherent
No change	2 (11)	10 (56)
Changed once	4 (22)	0
Changed twice	1 (6)	1 (6)
**Total**	**7 (39)**	**11 (61)**

Although most participants cited encouragement by doctors, pharmacists, family, friends and the media, only three participants specifically mentioned any sort of formal follow-up (one at a diabetic review, one with a pharmacist and one with a nurse). Many felt specific follow-up would have given them more confidence:

I mean I'm all for taking it if I know what it's doing and if it's doing you good, but there's no follow up on these things. I have to go and get my blood pressure taken every six months. I feel like they know what's going on cos they have changed the different strengths at different times you know depending on how my blood pressure is. But you don't get any follow up with this…. I think they need to have more follow up on this. I'm taking it because it's doing me what it's supposed to be doing, but it's the not knowing and it's the not having the follow up to see whether you need to be taking it or not. (Participant 11, age 77 – Continually Adherent Bisphosphonates. Calcium not prescribed)

One participant explicitly changed her mind and became adherent by Phase 2 having previously rejected the medication because of her doctor's insistence:

I didn't used to take that but then once I had this polymyalgia and I had to take the steroids. As I walked out of the doctors' room she said ‘now look you must take that acid tablet because if not your bones will just crumble’. So whether I like it or not I'm taking it. (Participant 13, age 75 – became adherent by Phase 2. Adherent to Calcium)

In summary, many participants saw the medication as an adjunct to their own efforts to remain healthy and ward off the impacts of ageing. Additional convenience of medication dosing, a less off-putting name, and ways to reduce side effects would be likely to positively influence people's decisions to remain adherent to these medications.

## Discussion

The data overall show a group of resilient older women doing their best to make sense of a particular set of health opportunities in their lives, and keen to manage the impacts of ageing and minimise increasing frailty and dependence. There was evidence that those who had engaged with professionals to establish and maintain regimens could tolerate and overcome barriers to adherence which had defeated others. The variation of adherence over time suggests that health professionals should not assume adherence in any older women who has relevant medication on their repeat medication list, and that the uncertainty of risk and desire not to worry participants must not confuse messages that these drugs can have a real positive health gain.

To our knowledge, no previous studies have undertaken longitudinal in depth interviews to identify factors which influence adherence to osteoporosis regimens in women who have been identified at high risk of fracture. Thus, the strength of this study is the repeat in depth interviews with older women that enabled follow up and discussion of change overtime. The limitations include potential sample bias: women who are keen to help research are potentially more motivated to self-care and take an active part in their own health.

However the context of this work is multifaceted and reflects the issues that arise following screening in a cohort of women who have not previously sought advice about their bone health. Overtime many medications that are routinely prescribed become objects embedded within everyday life and invested with particular meanings, values and identities [19]. However, our findings underline the fact that medications are complex social phenomena that may take time to embed and become routinized and acceptable to patients and healthcare professionals alike [20]. Medications, particularly medication prescribed prophylactically, have many levels of meaning [21]. Bisphosphonate medication for the prevention of osteoporosis and fracture is currently framed by a more complex social context where understanding, choice, risk and perceived need all interact to produce unpredictable patterns of usage and acceptability [22].

This study did not record consultation data or the perspective of general practitioners on their interactions around decision making on bisphosphonates. In depth sociolinguistic research has highlighted that patients talk and deploy different discourses and narratives depending on both the context and on the interactional resources available to them [23], [24], [25], [26]. Although most patients and their health care providers are likely to be keen to ensure that they utilise the outputs of modern medical advances, this is in the context of the increasing biomedicalisation of life, especially as people in developed countries are living longer [27]. Whether this is framed as a sophisticated commercial exploitation of societal fears of frailty and death, or as an unpredicted consequence of applying multiple disease related guidelines without considering the overall impact, there are consequences for individuals in making choices about their health-related practices each day. For example, recent authors have pointed out the likelihood of older people routinely being in receipt of multiple medications, even if they have not yet developed any specific disease [28], [29]. The cumulative costs of screenings, follow-ups, treatments and personal sequelae in terms of time, side effects, and perceived vulnerability or risk all need to be set against the potential benefits. The impacts of knowing that they are deemed to be at risk can move people from a narrative of their self as a healthy empowered person to one who is frail and in need of help, which can also have unexpected consequences [27].

Within this complex context, each patient and their doctor or nurse has to make individual decisions as to whether a risk factor should be prioritised or treated once detected. General practitioners are probably the medical professionals who are most aware of the extent to which the uptake of any new test or treatment is dependent on the beliefs, understanding, needs and expectations of the individual in front of them. It is a tenet of their discipline and training that any new health issue needs to be debated in the context of the person's whole life context, while maximising health gain and minimising adverse consequences of any intervention. It is the skill of a general practitioner to enable patients to make considered judgements in the face of these multiple choices. However, both the literature and our interviews suggest that preventive health measures often pose a challenge in time limited appointments, which may have influenced the decision made in some clinical encounters [30], [31], [32]. Furthermore, the fact that this was a research study may also have influenced overall uptake and follow-up in clinical practice. More research is needed on the effectiveness and efficacy of secondary prevention and the role of healthcare providers in this field.

## References

[1] Kanis JA (2002) Diagnosis of osteoporosis and assessment of fracture risk. Lancet 359: 1929–1936. 12057569 10.1016/S0140-6736(02)08761-5

[2] WHO (2003) Prevention and Management of osteoporosis. Geneva: WHO.

[3] Verdijk NA, Romeijnders AC, Ruskus JJ, Van Der Sluijs C, Pop VJ (2009) Validation of the Dutch guidelines for dual X-ray absorptiometry measurement. The British Journal of General Practice 59: 256–260. 19341554 10.3399/bjgp09X420338 PMC2662101

[4] (SIGN) SIGN (2003) Management of osteoporosis risk.

[5] NICE (2012) Osteoporosis: Assessing the risk of fragility fracture London: NICE. 32186835

[6] WHO Fracture Risk Assessment Tool.

[7] Kanis J, Glüer CC (2000) An update on the diagnosis and assessment of osteoporosis with densitometry. Osteoporosis International 11: 192–202. 10824234 10.1007/s001980050281

[8] Marshall IJWC, McKevitt C (2012) Lay perspectives on hypertension and drug adherence: systematic review of qualitative research. British Medical Journal 345. 10.1136/bmj.e3953 PMC3392078 22777025

[9] Solomon DHAJ, Katz JN, Finklestein JS, Arnold M, Polinski JM, et al (2005) Compliance with osteoporosis medications. Arch Intern Med 165: 2414–2419. 16287772 10.1001/archinte.165.20.2414

[10] Recker RRGR, MacCosbe PE (2005) Effect of dosing frequency on bisphosphonate medication adherence in a large longitudinal cohort of women. Mayo Clin Proc 80: 856–861. 16007889 10.4065/80.7.856

[11] Rossini MBG, Di Munno O, Giannini S, Minisula S, Sinigaglia L, et al (2006) Determinants of adherence to osteoporosis treatment in clinical practice. Osteoporosis International 17: 941-921. 10.1007/s00198-006-0073-6 16538553

[12] (IOF) IOF (2005) The adherence gap: Why osteoporosis patients don't continue with treatment Nyon: IOF.

[13] Pound PBN, Morgan M, Yardley L, Pope C, Daker-White G, et al (2005) Resisting medicines: A synthesis of qualitative studies of medicine taking. Social Science & Medicine 61: 133–155. 15847968 10.1016/j.socscimed.2004.11.063

[14] Brookhart MAAJ, Katz JN, Finklestein JS, Arnold M, Polinski JM, et al (2007) Gaps in treatment among users of osteoporosis medications: the dynamics of noncompliance. Am J Med 120: 251–260. 17349448 10.1016/j.amjmed.2006.03.029

[15] Pickney CSAJ (2005) Correlation between patient recall of bone densitometry results and subsequent treatment adherence. Osteoporosis International 16: 1156–1160. 15744452 10.1007/s00198-004-1818-8

[16] L. S A Pragmatic Randomised Clinical Trail of the Effectiveness and Cost Effectiveness of Targeted Population Screening for Low Bone Mineral Density in the Prevention of next of Femur Fractures: ISRCTN 11021925.

[17] Shepstone LFR, Lenaghan E, Harvey I, Cooper C, Gittoes N, et al (2012) A pragmatic randomised controlled trial of the effectiveness and cost-effectiveness of screening older women for the prevention of fractures: Rationale, design and methods for the SCOOP study. Osteoporosis International 23: 2507–2515. 22314936 10.1007/s00198-011-1876-7

[18] Ritchie J LJ (2003) Qualitative research practice: A guide for social science students and researchers. London: Sage Publications.

[19] Hodgetts D, Chamberlain K, Gabe J, Dew K, Radley A, et al (2011) Emplacement and everyday use of medications in domestic dwellings. Health & Place 17: 353–360. 21163684 10.1016/j.healthplace.2010.11.015

[20] Cohen D, McCubbin M, Collin J, Pérodeau G (2001) Medications as social phenomena. Health 5: 441–469.

[21] Shoemaker SJ, Ramalho de Oliveira D, Alves M, Ekstrand M (2011) The medication experience: Preliminary evidence of its value for patient education and counseling on chronic medications. Patient education and counseling 83: 443–450. 21435815 10.1016/j.pec.2011.02.007

[22] Brown P, Calnan M (2012) Braving a faceless new world? Conceptualizing trust in the pharmaceutical industry and its products. Health 16: 57–75. 21169205 10.1177/1363459309360783

[23] Murdoch J, Salter C, Cross J, Smith J, Poland F (2013) Resisting medications: moral discourses and performances in illness narratives. Sociology of Health & Illness 35: 449–464. 22742778 10.1111/j.1467-9566.2012.01499.x

[24] Salter C, Holland R, Harvey I, Henwood K (2007) “I haven't even phoned my doctor yet.” The advice giving role of the pharmacist during consultations for medication review with patients aged 80 or more: qualitative discourse analysis. BMJ 334: 1101. 17449504 10.1136/bmj.39171.577106.55 PMC1877907

[25] Britten N, Stevenson F, Gafaranga J, Barry C, Bradley C (2004) The expression of aversion to medicines in general practice consultations. Social science & medicine 59: 1495–1503. 15246177 10.1016/j.socscimed.2004.01.019

[26] Murdoch J, Salter, C., Cross, J., & Poland, F. Misunderstandings, communicative expectations and resources in illness narratives: Insights from beyond interview transcripts. 10.1558/cam.v10i2.153 24851510

[27] Salter CIHA, Howe A, McDaid L, Lenaghan E, Blacklock J, et al (2011) Risk, significance and biomedicalisation of a new population: Older women's experience of osteoporosis screening. Social Science & Medicine 73: 808–815. 21813219 10.1016/j.socscimed.2011.06.030

[28] Moynihan RDJ, Henry D (2012) Preventing overdiagnosis: How to stop harming the healthy. British Medical Journal 244. 10.1136/bmj.e3502 22645185

[29] Moynihan RHI, Henry D (2002) Selling sickness: The pharmaceutical industry and disease mongering. British Medical Journal 324: 886–891. 11950740 10.1136/bmj.324.7342.886 PMC1122833

[30] Mirand ALBG, Kuo CL, Mahoney MC (2003) Explaining the de-prioritization of primary prevention: Physicians' perceptions of their role in the delivery of primary care. BMC Public Health 3: 1–15. 12729463 10.1186/1471-2458-3-15 PMC155789

[31] Williams SJCM (2008) Perspectives on prevention: The views of general practitioners. Sociol Health Ill 16: 372.393.

[32] Poole KESCJ (2006) Osteoporosis and its management. BMJ 333. 10.1136/bmj.39050.597350.47 PMC1702459 17170416

